# School nutrition programs in Dubai: a landscape analysis

**DOI:** 10.3389/fpubh.2025.1584497

**Published:** 2025-07-25

**Authors:** Reem AlGurg, Nour Abu Mahfouz, Farah Otaki, Agnes Paulus, Amar Hassan Khamis, Mohamad Alameddine

**Affiliations:** ^1^Strategy and Institutional Excellence, Mohammed Bin Rashid University of Medicine and Health Sciences, Dubai, United Arab Emirates; ^2^College of Medicine, Mohammed Bin Rashid University of Medicine and Health Sciences, Dubai, United Arab Emirates; ^3^Department of Health Services Research, Faculty of Health, Medicine, and Life Sciences (FHML), Care and Public Health Research Institute (CAPHRI), Maastricht University, Maastricht, Netherlands; ^4^School of Health Professions Education (SHE), Faculty of Health, Medicine, and Life Sciences (FHML), Maastricht University, Maastricht, Netherlands; ^5^Hamdan Bin Mohammed College of Dental Medicine, Mohammed Bin Rashid University of Medicine and Health Sciences, Dubai, United Arab Emirates; ^6^Department of Health Care Management, College of Health Sciences, Research Institute of Medical and Health Sciences, University of Sharjah, Sharjah, United Arab Emirates

**Keywords:** school nutrition programs, nutrition, school-aged children, healthy living, healthy school, sustainable development, sustainable development goals, SDG 3, 4, 9 and 17

## Abstract

**Background:**

Little is known about the execution of School Nutrition Programs (SNPs) within schools across the public and private sectors in Dubai, United Arab Emirates. This highlights the importance of capturing an inside perspective about the specificities of the SNPs in order to ensure their effective, efficient, and equitable implementation across schools, irrespective of whether they are public or private.

**Objective:**

The overall purpose of this study was to develop insight into SNPs in Dubai, and to investigate the difference in characteristics of those SNPs, between the public and private sectors.

**Methods:**

This study relied on a quantitative tailor-made survey. The data were analyzed using SPSS; descriptive analysis consisted of computing the proportions for all the variables. In terms of inferential analysis, chi-square test of independence was selected to determine whether there are associations between the categorical variables (i.e., the various characteristics of the SNPs, and whether the corresponding schools are public or private).

**Results:**

Out of 75 school representatives who were invited to participate in the current study, 60 responded, of whom 48 school representatives indicated that their respective schools had SNPs. These 48 SNPs had varying implementation scopes and program compositions (i.e., combination of initiatives) of Parents’ Involvement, Lunch Box, Educational Curriculum, Hydration, Awareness Activities, School Canteen, and/ or Food Safety and Hygiene. The stakeholders involved in developing and maintaining the respective SNPs and the intended outcomes of those programs also differed across the schools.

**Conclusion:**

This study highlights, in alignment with Sustainable Development Goals 3, 4, 9 and 17, the importance of the proposed reformation around SNPs in Dubai to take into account the governance structure on the local and national levels, quality assurance measures, stakeholder engagement, and programs’ intended outcomes and compositions. It proposes the enactment and maintenance of holistic, school-level healthy living programs that include nutrition as part of a more comprehensive approach to fostering the students’ individual and collective wellbeing.

## Introduction

School Nutrition Programs (SNPs) are structured initiatives aimed at providing balanced meals to school-age children, significantly contributing to their health, educational outcomes, and overall wellbeing. The exact meaning of SNPs and what they encapsulate tend to differ between countries. They are often part of some sort of a more holistic “healthy school” programs (including but not limited to nutrition) (1–4). These programs, irrespective of their implementation scopes and compositions, are recognized as vital components of national development strategies, particularly in low-income countries where they often rely on international donor support (5). The link between the SNPs and the increase in wellbeing is well established (6). Moreover, it is proven that focusing policies at a school level on such endeavors can reap substantial health benefits in this demographic. International nutrition-related organizations strongly recommend that schools have strong and comprehensive nutritional programs, and promote nutrition education, as well as strengthen school-home-community partnerships, to maximize the positive impact on the general health of the community (7–9).

SNPs serve not only to combat malnutrition but also to enhance school attendance and performance, as evidenced by various studies indicating positive impacts on enrollment and completion rates (10–13). It is established that nutrition directly influences students, including but not limited to their school performance (14–17). Nutrition and wellness programs and plans in schools influence children in and out of school’s eating habits (18, 19). A study conducted on 1,178 children in primary schools in different urban cities in the United States measured the effect of school-based nutritional intervention. The respective study concluded that these interventions lead to an immediate effect on children’s Body Mass Index (BMI), hence influencing their susceptibility to non-communicable diseases (20).

Furthermore, the evolving objectives of the corresponding programs now encompass broader goals, including promoting sustainable food systems and supporting local agriculture (21). SNPs are essential for fostering healthier future generations and addressing systemic issues in food security and education (22). SNPs not only affect children but also have an impact on their families as shown in a study conducted in Iran to evaluate the implementation of a national nutrition program for improving nutritional status of children. It showed that the program had great impact on enhancing mother’s knowledge on health, nutrition, and child care (23). Another study that involved 50 semi-structured interviews with school principals and teachers concluded that school nutrition policies and guidelines are significant to enhance students’ healthy eating which directly affects the students’ cognitive functioning and in turn their academic performance. Many programs struggle to maintain momentum beyond initial funding, necessitating strong community involvement and adaptive strategies to ensure long-term success (24). Thus, a multifaceted approach that engages various stakeholders is essential for the sustainability and effectiveness of school nutrition programs (24, 25). Even if the corresponding polices are mandated, their effect on children health and their BMI is highly dependent on their effective, efficient, and equitable implementation (26, 27), which ultimately relies on the competencies (i.e., knowledge, skills, attitudes, and habits) of the school-level school nutrition program personnel and their modus operandi (16, 28–30).

The first part of this multi-staged research work systematically analyzed the perception of key opinion leaders, positioned at differing levels of the public health and education ecosystems in the United Arab Emirates (UAE). It showcased the macro-perspective of the planning, implementation, and evaluation of SNPs in Dubai, UAE (27). Little is known, though, about what is actually happening, in terms of SNPs execution (including: school food/ nutrition policy and objectives, implementation scopes, compositions, and monitoring and evaluation schemes), within the respective schools, across the public and private sectors. This highlights the importance of capturing an inside perspective about the specificities of the SNPs in order to ensure effective, efficient, and equitable implementation across schools, irrespective of whether they are public or private. Hence, the overall purpose of this study was to develop insight into the SNPs in Dubai, and to investigate the difference in characteristics of those SNPs, between the public and private sectors. As such, this study addresses the following research questions, from the perception of school representatives:

- What is the status quo of SNPs in Dubai?
- How does the implementation of SNPs differ between the public and private schools in Dubai?

## Materials and methods

### Context of the study

This study took place in Dubai, UAE in the academic year 2018–2019. With more than 3 million citizens (a third of the country’s population), Dubai is the most populated Emirate of the UAE (31). Sustaining and promoting public health in the UAE is one of the top priorities of the country’s leaders, where they aim to have more active and healthy citizens supporting the development of the country. As a result, one of the main parts of the UAE national agenda is non-communicable diseases (NCDs) prevention. This goal has been put into focus earlier during the high general United Nations (UN) assembly in 2011 which cascaded down to World Health Organization (WHO) guidelines internationally, in the Middle East and North Africa region (MENA), and into the Gulf countries (32–34). A systematic review identified 298 government-led, school-based nutrition programs in MENA referred to in the literature that is published between 2001 and 2021. The most common interventions were school meals and school feeding programs, followed by nutrition education within the curriculum, extracurricular nutrition education, standards for school canteens or foods/beverages available in schools, and training of school staff (32). Another systematic review that scanned the available scientific evidence pertaining to school-based nutrition interventions across MENA revealed that appropriately designed and implemented nutrition interventions has been positively impacting nutrition knowledge, and health and diet-related behaviors in children and adolescents in MENA (35). A lot of work has been done in the UAE regarding NCDs, the UAE’s vision 2021 aims to achieve a world-class healthcare system and entails a decrease in lifestyle diseases as part of the strategy for the nation.

One of the health indicators of the UAE is the prevalence of obesity among children which has been adopted from the corresponding WHO framework. This indicator measures the proportion of children between the ages of 5 and 17, out of the total children of the same age group, who are considered obese. In the academic year 2017–2018, based on the output of analysis of the National Health Survey, the obesity rate among children and adolescents aged between 5 and 17 years reached about 17.35 percent. The set target of the national agenda was to reduce this value to 12% by the year 2021. Unfortunately, the respective value rose in 2020, which can be due to the lifestyle restrictions that accompanied Coronavirus Disease of 2019 (COVID-19) (36). At the time of the current study, in the academic year 2018–2019, Dubai reported 280,979 registered students in private schools and 29,387 students in public ones (31). The drive behind pursuing the dissemination of the findings generated from the analysis of data collected right before the onset of COVID-19 is to call for the reprioritization of improving the health and wellbeing of children through evidence-driven school interventions.

Multiple federal and Emirate level governing bodies have been working toward lowering the value of this indicator and have generated school nutrition guidelines. These entities include Ministry of Health and Prevention (MOHAP), Department of Health- Abu Dhabi, Dubai Health Authority (DHA), Dubai Municipality, and Ministry of Education (MOE) (37). The UAE has a national Nutrition Strategy that has a few subsections related to schools, but does not have a national policy specific for school nutrition (38). Relevantly, to the best of the authors’ knowledge, there is no single definition of SNPs in the context of Dubai and/ or UAE. Although all the relevant entities are working to reduce the childhood obesity prevalence, there remains to be discrepancies across the guidelines that are influencing the planning, implementation, and monitoring and evaluations of SNPs (27). Relevantly, there has been several press releases that reflect key stakeholders’ intentions to direct concerted efforts toward promoting healthy and sustainable nutrition in UAE schools (39, 40). For example, several DHA stakeholders around September 2014, unofficially communicated that all schools in the UAE are required to follow healthy nutritional guidelines starting the respective academic year (40). More recently, around September 2024, school nutrition guidelines set by the abovementioned authorities were informally brought-up again by Education UAE magazine (39). According to the respective news article, these guidelines are designed to promote healthy eating habits among children, reduce obesity, and accommodate allergies and other dietary needs. This news article also highlights that the corresponding requirements cover nutritional value, sustainable and safe food, and addressing allergies. It seems that the existing guidelines, albeit valuable, are not necessarily comprehensive in scope, in terms of healthy living, or part of a more holistic approach to school-level health and wellbeing programs.

### Research design

**Figure 1:**
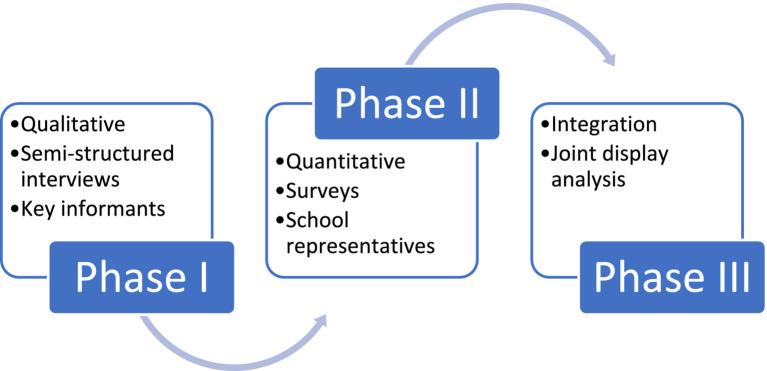
Outline of the multi-staged research work that the current study (phase II) is a part of.

The current study is the second part of a multi-staged research work, which is composed of three phases and relies on a convergent mixed methods research design (41) to enable the development of systemic understanding of SNPs in Dubai (42) (Figure 1). Ethical approval for this research work was obtained from the International Review Board (IRB) at the Mohammed Bin Rashid University of Medicine and Health Sciences (MBRU), Dubai, UAE, under the reference number MBRU-IRB2018-029.

The first phase of the research work was qualitative and revolved around conducting semi-structured interviews with a diverse group of key informants to explore their perceptions in relation to the subject matter (27). As for the current study, it is quantitative and aims at describing the school representatives’ perception of status-quo of SNPs in Dubai, and how (from the point-of-view of the school representatives) the public and private schools differ from one another, regarding SNPs. The last phase of this multi-staged research work will be around systematically integrating the findings of the two preceding phases using joint display analysis process (43).

### Data collection

A tailor-made survey was developed for the purpose of the current study. The survey’s content and structure was based on prior research, and inspired by several other data collection tools that were previously deployed, across different countries around the world (e.g., “foods and physical activity in public elementary schools” developed and first assembled in 2005 by the United States Department of Education National Center for Education Statistics Washington, D. C.), to systematically capture and investigate the views of stakeholders on school food systems (44–47). Before starting the data collection, the survey was validated. This validation entailed randomly selecting 10 private and 10 public schools, and inviting them to provide their feedback on the respective survey for both content (i.e., to assess the extent to which the survey covers all relevant parts of the construct it is meant to measure) and face validity (i.e., to assess the clarity, comprehensibility, and readability of the questions, and the flow by which they are presented). Out of the 20 invited schools, 8 expressed openness to engage in the validation phase (the rest either did not respond or expressed that they would not be available to engage in this phase given time constraint), out of which 5 actually provided their feedback either verbally, over the phone, or through an email (the remaining three did not respond). The gathered feedback led to modifying the categories of a few of the variables, including adding potential initiatives/ activities to the multi-select question that inquires about the implementation scope and program composition.

Based on Cochrane sample size calculation, it turned out that 59 schools are needed to participate in the study to have a confidence level of 95%. To ensure reaching the calculated sample size, the researchers chose to aim for 18% more schools than the required sample size indicated by the calculation (expected nonresponse rate), and hence the target sample size became 75. Accordingly, 75 schools (21 public and 54 private) were selected using Stratified Probabilistic Sampling technique, where the distribution of schools in 2018 in Dubai, according to Dubai Statistics (48), was 75% private and the rest were public (*N* = 280). It is worth noting that the schools which were selected for the validation phase (i.e., a total of 20) were excluded from the study participants’ randomization. The informed representatives who were nominated by the leadership teams of the selected schools to fill the electronic survey (Appendix I), on behalf of the respective schools, were asked to provide an electronic informed consent prior to participating in the study. There were English and Arabic versions of the survey.

The survey was composed of three segments. The first segment inquired for general information about the school including its location; whether it is public or private; curriculum; grades; whether it is female, male, mixed, or gender-isolated; and number of students. Then, in the second segment, there were questions related to the demographics of the participants (i.e., school representatives) filling in the electronic survey including their respective job title, department, gender, nationality, and years of experience in the education system in the UAE. The last segment of the survey contained 13 questions, starting with inquiring whether, or not, the respective school has a school nutrition program, followed by 10 inquiries of the descriptions of the school nutrition programs (including: school food/ nutrition policy, stewardship, oversight, and program implementation scope, responsibilities, outcomes, structure, scale, and monitoring and evaluation). The last two questions were meant to be answered by the school representatives, only if they indicate in response to the first question of this segment that their respective school does not have a school nutrition program.

The data was collected between April and August 2019. All the schools that were randomly selected were contacted through a phone call to get the email address of the school principal, or that of a representative from within the respective schools nominated (by the respective school leadership team) to provide their feedback about the schools’ nutrition practices. The electronic survey was then sent to those email addresses. Links to Arabic and English versions of the survey were embedded in the emails. The informed school representative had the option to choose their preferred language. There were three reminders (1 month apart) by email and/ or phone calls to encourage the select school representative to take part in the study by responding to the electronic survey.

### Data analysis

The quantitative data were analyzed using SPSS for Windows version 27. The descriptive analysis consisted of computing the proportions for all the variables.

In terms of the inferential analysis, chi-square test of independence was selected to determine whether there are associations between the categorical variables (i.e., the various characteristics of the SNPs, and whether the schools are public or private).

## Results

Out of 75 school representatives who were invited to participate in the current study, as per Table 1, 60 responded (i.e., overall response rate = 0.80).

**Table 1:** Response rates across public and private sectors.

Sector	Number of responses	Total number of schools	Response rate
Public	19	21	0.90
Private	41	54	0.76
Total	60	75	0.80

As such, 19 (31.7%) of the participants were representatives of public schools and 41 (60%) were representing the private sector. In terms of the school curriculums, 48.3% were Ministry of Education (all of the public schools and 11 of the private schools), 18.3% were British, 13.3% were American, 5% were from each of Indian and Iranian, 3.3% were from each of International Baccalaureate and Pakistani, and 1.7% from each of French and Japanese. Out of 60 participating schools, 55% had Kindergarten, 58.3% had Primary, 60% had Intermediate, and 57.6% had Secondary levels (Table 2).

**Table 2:** Distribution of schools covering respective levels of learning across public and private sectors.

Levels	Public	Private	Total
Kindergarten	4	29	33
Primary	4	31	35
Intermediate	6	30	36
Secondary	9	25	34

Most of the schools were mixed 32 (53.3%), 28 (87.5%) of which were Private and 4 (12.5%) of which were Public. The remaining schools were males’ schools (28.3%- 9 public and 8 private), females’ schools (10%- all of which are public), and gender-isolated schools (8.3%- all of which are private). In terms of the number of enrolled students, 29 (48.3%) schools had up to 500 students, 15 (25%) schools had between 501 and 1,000 students, and another (15) 25% schools had between 1,001 and 3,000 students, and only 1 (1.7%) school had more than 3,000 students (Table 3).

**Table 3:** Distribution of schools’ number of students across public and private sectors.

Number of students	Public	Private	Total
Up to 500 students	13	16	29
501 through 1,000	5	10	15
1,001 through 3,000	1	14	15
More than 3,000	0	1	1
Total	19	41	60

In terms of the characteristics of the school representatives (who responded to the survey- i.e., current study participants), 31 (51.7%) were School Principals, 7 (11.7%) were Head of Administration, 4 (6.7%) were Health and Safety Administrators, 3 (5%) were School Doctors, 10 (16.7%) were School Nurses, and 5 (8.3%) were Activities Coordinators. These representatives were mainly members of School Administration: 33 (55.4%). The rest were members of Health Department 13 (21.4%), Academics 12 (19.6%), and Catering Departments 2 (3.6%). Out of the 60 representatives, 41 (68.3%) were female and the rest were male 19 (31.7%), and 41 (68.3%) were non-Emiratis and the rest were Emiratis: 19 (31.7%). In relation to their years of experience, 22 (36.7%) had up to 5 years, 7 (11.7%) had between 6 and 10 years, and 31 (51.7%) had more than 10 years of experience.

Among the 60 participating schools, 48 (80%) had SNPs [13 (27.1%) public and 35 (72.9%) private] and 12 (20%) did not have SNPs. For those 48 schools with SNPs, 31 (64.6%) were governed primarily by private policy, and the rest [17 (35.4%)] relied mainly on national policy, with no statistically significant difference between public and private sectors (Table 4).

**Table 4:** Distribution of schools’ primary governing body across public and private sectors.

Type of policy	Public schools	Private schools	Total
National policy	2	15	17
Private policy	11	20	31
Total	13	35	48

Regarding the entities outside the respective schools who are considered responsible of the SNPs, 18 (37.5%) school representatives indicated the Ministry of Education [this was the case significantly more in the public sector relative to the private sector (*p* = 0.002)], 24 (50%) indicated Dubai Health Authority [this was the case significantly more in the private sector relative to the public sector (*p* = 0.008)], and 27 (56.3%) indicated Municipality [this was the case significantly more in the private sector relative to the public sector (*p* = 0.049)]. As for the rest, 1 private school seemed to resort to the British Curriculum only and 2 private schools seemed to turn to their internal health regulations.

Also, out of the 48 SNPs, 25 (52.1%) included Parents’ Involvement (with no statistically significant difference between public and private sectors), 28 (58.3%) included Lunch Box [this was the case significantly more in the private sector relative to the public sector (*p* = 0.001)], 30 (62.5%) included Educational Curriculum (with no statistically significant difference between public and private sectors), 32 (66.7%) included Hydration (with no statistically significant difference between public and private sectors), 37 (77.1%) included Awareness Activities [this was the case significantly more in the private sector relative to the public sector (*p* = 0.047)], 39 (81.3%) included School Canteen (with no statistically significant difference between public and private sectors), and 39 (81.3%) included Food Safety and Hygiene [this was the case significantly more in the private sector relative to the public sector (*p* = 0.048)] (Table 5).

**Table 5:** Scope/composition of SNPs in schools across public and private sectors.

Scope/composition	Public schools	private schools	Total
Parents’ involvement (*p* = 0.335)	5 (20%)	20 (80%)	25 (52.1%)
Lunch box (*p* = 0.001)	2 (7.1%)	26 (92.9)	28 (58.3%)
Educational curriculum (*p* = 0.190)	6 (20%)	24 (80%)	30 (62.5%)
Hydration (*p* = 0.310)	7 (21.9%)	25 (78.1%)	32 (66.7%)
Awareness activities (*p* = 0.047)	7 (19%)	30 (81%)	37 (77.1%)
School canteen (*p* = 0.687)	10 (25.64%)	29 (74.36%)	39 (81.3%)
Food safety and hygiene (*p* = 0.048)	8 (20.51%)	31 (79.49%)	39 (81.3%)

In 34 (70.8%) of the 48 schools, the Medical Staff were involved in developing and maintaining SNPs [this was the case significantly more in the private sector relative to the public sector (*p* = 0.034)], in 29 (60.4%) of them, Administrative Staff were involved [this was the case significantly more in the private sector relative to the public sector (*p* = 0.07)], in 24 (50%) of them, Academic Staff were involved [this was the case significantly more in the private sector relative to the public sector (*p* = 0.049)], in 18 (37.5%) of them, Parents were involved (with no statistically significant difference between public and private sectors), and in 14 (29.2%) of them (all private), the School Nutritionists were involved.

In terms of program outcomes, most of the SNPs aimed at preventing and/ or reducing obesity: 45 (93.5%), improving nutrition: 43 (89.1%), preventing and/ or reducing malnutrition: 30 (63%), and improving students’ academic performance: 25 (52.2%). Also, half of the SNPs included: supporting parents and the local community, among their outcomes. Only 18 (37%) of the SNPs had: addressing health inequalities, and 17 (34.8%) of the SNPs had: improving students’ school attendance among their outcomes. There appeared to be no statistically significant difference between private and public sectors across all the above mentioned program outcomes. The only exception was: “supporting parents and the local community,” where it was significantly more likely to be indicated as an outcome in private schools (*p* = 0.47).

Also, 87.5% of the SNPs had measures in place to ensure implementation (i.e., 39 out of the 48 SNPs) with no statistically significant difference between the sectors, out of which 31 SNPs (64.6%) had measures in place to assure the quality of implementation (with defined performance indicators) with no statistically significant difference between the sectors. Most of the SNPs include breakfast: 39 (81.3%) and snacks: 26 (54.2%). Half of the SNPs include lunch, and only 5 (10.4%) include dinner. All the SNPs that include the lunch meal belong to the private sector. As for the rest of the meals, there seemed to be no statistically significant difference in their distribution between the sectors.

The SNPs covered Kindergarten in 27 (56.3%) of the 48 schools [this was the case significantly more in the private sector relative to the public sector (*p* = 0.008)], Primary in 29 (60.4%) [this was the case significantly more in the private sector relative to the public sector (*p* < 0.001)], Intermediate in 27 (56.3%), and Secondary in 19 (39.6%). There seemed to be no statistically significant difference between SNPs coverage of the latter two categories of learning levels between private and public sectors.

For the 12 represented schools that reported not having SNPs, 11 (91.7%) were aware of the school nutrition guidelines in Dubai, and 7 (58.3%) had a plan to devise SNPs in the coming 3–5 years, with no statistically significant difference between sectors.

## Discussion

The current study showed that most of the participating representatives belong to schools that have SNPs (80%). Almost all of the participating schools without SNPs (except for one) seemed to be aware of school nutrition guidelines in Dubai, and the majority of them had a plan to devise SNPs in the near future. Almost two-thirds of those SNPs were governed primarily by private policy. There appeared to be evident variation in what school representatives indicated as external entities responsible for overseeing SNPs. This could be because of poor awareness among the participating school representatives, and/ or due to multiplicity of guidelines (issued by different stakeholders), limited coordination across governing entities (local and federal), and/ or lack of clarity regarding the roles and responsibilities of relevant external local and federal entities. This calls for forming a unified governing body around SNPs in Dubai, in alignment with the United Nations Sustainable Development Goals 3, 4, and 9, and 17 (49, 50), where effective collaboration across all involved entities is maximized. This governing body should make having SNPs mandatory across all public and private schools in Dubai. This needs to be compounded with clear communication with all relevant stakeholders of the exact roles and responsibilities of the entities involved to raise efficiency and attain effective interdependencies. In one of the previously mentioned systematic reviews, which scanned the literature for government-led school nutrition programs across MENA, highlighted that 13 countries have reported the establishment of standards for school canteens, and/ or for foods and beverages available in schools and kindergartens. The highest number of reported programs were from Gulf Cooperation Council countries (GCC), including Bahrain and Kingdom of Saudi Arabia, as well as Iran. According to that review, some countries have also reported on standards related to food vendors and outlets around the schools (32). Relevantly, in Egypt, the 100 million Health Initiative included the provision of outlets for selling healthy and safe food in the vicinity of schools and public places (51). There is also IRAN-Ending Childhood Obesity program, which aimed at limiting the availability of unhealthy snacks and fast foods by street food vendors around the schools (52, 53). Along those lines, in Japan, in order to ensure standardization of in-school, nutrition-related activities, the government have enacted laws that schools need to abide to. For example, the “School Lunch Program Act” requires all schools to provide safe and well-balanced school lunches to students (54). Similarly, in Korea, two laws: Special Act on Children’s Dietary Life Safety Management of 2008 and Dietary Life Education Support Act of 2009, led to the standardization of meaningful changes in school nutrition environment and practices (55). As such, a unified implementation plan needs to be developed and implemented across all schools in Dubai. Ideally, this plan should be holistic, handling nutrition as a segment of more comprehensive school-level programs that aim to promote healthy living. In fact, the unified implementation plan can characterize (within the context of Dubai, in specific, and the UAE more generally) the concept of a “healthy school” (56–58) which promotes students’ wellbeing (including but not limited to fostering physical activity, and securing access to physical and behavioral health services, and to healthy spaces to learn and play), and require and incentivize all schools to continuously develop in that direction.

Moreover, empowering the relevant administrators within the schools is also important to maximize buy-in and effective engagement. This can be done through capacity building. Evidence from MENA shows that teachers trained by experts such as dietitians and pediatricians are effectively implementing nutrition interventions that achieve the intended outcomes (35). A particularly useful set of skills, along with awareness of the intricacies of the proposed Emirate-level implementation plan, would be through the attainment and maintenance over time of Project Management Professional (PMP)® certificates (59). Proficiently handling any one school nutrition program as a set of interdependent projects [i.e., a “program,” according to the Project Management Body of Knowledge (PMBOK)®], as opposed to just a set of ongoing operations, is likely to raise the quality of its implementation and the likelihood of attaining its strategic goals (60). The projects can be traditional (i.e., predictive), where their scope is known and clearly articulated at the beginning, and/ or more agile (i.e., adaptive) progressively elaborating to absorb changes in scope as the implementation unfolds. The latter tends to be iterative/ incremental and require more interactivity. According to PMBOK, the entailed projects can also be a combination of “predictive” and “agile,” and hence, “hybrid” (61, 62). The corresponding project governance framework will need to outline the structure, people, and flow of information/ communication channels (60, 63).

The vast majority of the SNPs, represented in the current study, had measures in place to assure quality implementation. This needs to be compounded (and vertically aligned) with firm local and/ or federal performance measure and oversight (including but not limited to an overarching monitoring and evaluation framework), all of which will feed into the United Nations Sustainable Development Goals 3, 4, and 9, and 17 (64). Relevantly, in Finland, national and local regulations constitute the basis for school meal practices. Moreover, education acts and decrees in Finland, along with national core curricula and local curricula, are central documents governing school lunches. Finnish legislation guarantees students the right to free meals during school days, and the government guidelines offer detailed support for planning and serving school food (65).

The vast majority of SNPs, across the schools represented in the current study, seemed to involve medical and administrative staff. Half of the SNPs appeared to involve academic staff. SNPs, in the private sector, seemed to be more engaging, and approach matters in a more cross-disciplinary manner. There seemed to be an opportunity to increase engagement of parents in SNPs in both public and private schools, since it is established that direct parental involvement in SNPs significantly increase the programs’ impact in terms of improving the children’s weight status, physical activity, and/ or sedentary behavior (66). Previously conducted studies have repetitively alluded to importance of inclusion of a parent component in both treatment and prevention interventions to improve child weight status outcomes (67). This also applies to the engagement of school nutritionists in SNPs, where the opportunity appeared even greater among public schools. In this way, schools will leverage existing resources to address the suboptimal resourcing of SNPs challenge that was also identified as a major opportunity for improvement in the first part of this multi-staged research work (27).

The primary focus of the SNPs represented in the current study appeared to be on the breakfast meal. Only private schools appeared to include the lunch meal in their SNPs. It would be important to improve the inclusiveness of SNPs which was also flagged as a limitation, in the preceding part of this multi-staged research work (27), given the diverse student body and correspondingly varied health needs and cultural preferences. The current study also showed that the higher the grade (i.e., learning level), the less likely the SNPs coverage. The private sector schools seemed to be covering Kindergarten and Primary levels more than public sector. Most SNPs, across public and private schools, seemed quite diverse in terms of their composition (i.e., included activities), and the ones in the private schools seemed even more varied. This finding is not unexpected due to the level of autonomy given to private schools in relation to their health awareness programs, in general, and specifically to their SNPs.

The intended outcomes of the SNPs appeared to be quite diverse. There were no major differences in intended outcomes of SNPs between public and private sectors. Private sector seemed more considering of (and proactive about positively influencing) variables, affecting the effectiveness of the SNPs, external to respective schools (e.g., parents and local community). Moreover, it is established in the literature that food, nutrition, and dietary education programs leads to favorable results among children and adolescents, significantly improving their food consumption and diet quality (68, 69). Fortunately, a good proportion of the included SNPs included Educational Curriculum (62.5%) (with no statistically significant difference between public and private sectors). Along those lines, Japanese schools, as required by their government, emphasize education on food and nutrition. This is usually done through school educational activities. School lunches are served as a “living textbook,” where children are enabled to learn about various topics including but not limited to: food cultures and local traditional foods, eating manners, gratitude to those preparing meals, food production, and distribution and consumption. In Japan, as well, there are also numerous efforts made to enable children to acquire accurate knowledge about food and nutrition, good judgment on food choices, and a healthy and wholesome diet (55). As for the government of India, it gives a high priority to the issue of malnutrition among children. The Ministry of Education, across all the government and government-aided schools in India, provides nutritional meal to every child. The government also encourages the provision of nutritious and healthy food to children on special occasions and festivals. This led to the development of a feeling of equity and fostered a sense of belonging toward the community among the children (70).

This study is characterized by a few limitations. The generalizability of the findings is limited since the investigation was specifically around SNPs in Dubai. There is also a potential for social desirability bias, where the participants might have chosen to respond in a manner that is viewed favorably by others. In addition, the tailormade data collection tool, deployed for the current study, solicited the participants description of the SNPs using closed-ended questions, which limited the gathered details to existence/ absence of particular potential SNP components (i.e., Parents’ Involvement, Lunch Box, Educational Curriculum, Hydration, Awareness Activities, School Canteen, and Food Safety and Hygiene). Relevantly, the tool did not inquire about school meal diversity and consumption. Also, the tool validation phase involved inviting representatives from 10 public and 10 private randomly selected schools to offer their feedback. Ideally, the sampling strategy for the validation phase should have been stratified, as well, to be representative of the respective population. In addition, the low response rate of the validation phase (only five out of 20) restricted the generalizability of the obtained feedback (in terms of preparing and in turn endorsing the tool for the current study’s data collection). It would be worth following up this investigation with an exploratory lens to obtain more descriptions of the SNPs, and in turn develop a more thorough understanding of what is happening, in terms of SNPs, on-the-ground. Furthermore, it is important to develop a thorough understanding of student perceptions of the subject matter in order to inform consumer-centric policy reformations. It is recommended for future studies to capture the perspectives of the ultimate beneficiaries: the students and their families. It will also be useful to effectively integrate, via mixed methods research, quantitative with qualitative data to develop a holistic understanding of the status quo of the subject matter.

## Conclusion

This study builds on the preceding part of the current research work by calling for the formation of a unified governing body of SNPs in Dubai, in alignment with the Sustainable Development Goals. The proposed reformation is meant to mandate all schools in Dubai to have SNPs, putting equal emphasis on the public and private sectors. Moreover, this reformation would enable the upscaling of those SNPs through effective collaboration and co-creation. This study highlights the importance of, taking into account the governance structure on local and national levels; quality assurance measures; stakeholder engagement; and programs’ intended outcomes and compositions. It also emphasizes the added value of upskilling among school-level SNPs administrators with project management competencies to enable standardized handling of SNPs, each as a set of interdependent projects, which is expected to raise the quality of programs’ implementation and the likelihood of their attainment of strategic goals. Finally, this study proposes the enactment and maintenance of holistic, school-level healthy living programs that include nutrition as part of a more comprehensive approach to fostering the students’ individual and collective wellbeing.

## Data Availability

The original contributions presented in the study are included in the article/Supplementary material, further inquiries can be directed to the corresponding author.
