# Supplementary material for: School nutrition programs in Dubai: a landscape analysis
Source: Front Public Health. 2025 Jul 25;13:1584497. doi: 10.3389/fpubh.2025.1584497 (PMC12331509; doi:10.3389/fpubh.2025.1584497)
Supplement: Supplementary file 1 [file Supplementary_file_1.docx]

**Appendix I: Survey**

**Section 1: General Information**

- 1. School location (area) in Dubai: ____________________
  2. Please choose whether your school is:

1. Public
2. Private
   1. School curriculum:
3. Ministry of Education
4. British
5. American
6. Iranian
7. Indian
8. Other- please specify: _____________________
   1. Grades (select all those that apply):
9. Kindergarten
10. Primary
11. Intermediate
12. Secondary
    1. Gender of students:
13. Females’ school
14. Males’ school
15. Mixed school
16. Gender-isolated school
    1. Total number of students in school:
17. Up to 500 students
18. 501 through 1,000 students
19. 1,001 through 3,000 students
20. More than 3,000 students

**Section 2: School Representative Demographic Information**

2.1. Job title: _____________________

2.2. Department: ________________________

2.3. Gender:

1. Male
2. Female

2.4. Nationality:

1. Emirati
2. Non-Emirati

2.5. Years of experience in the educational sector in the UAE:

1. Up to 5 years
2. 6 through 10 years
3. More than 10 years

**Section 3: School Nutrition Program Information**

3.1. Do you have a nutrition program in place at your school?

1. Yes- answer questions 3.2. – 3.11.
2. No- answer questions 3.12. – 3.13.
   1. What regulates the nutrition program?
3. National policy
4. Private policy
5. Other -please specify: ___________________
   1. Which ministry or government entity is primarily responsible for the school nutrition program? (select all those that apply)
6. Ministry of Education
7. Ministry of Health and Prevention
8. Dubai Health Authority
9. Municipality
10. Other- please specify: ___________________
    1. What medium does the nutrition program cover? (select all those that apply)
11. Parents involvement
12. Lunch box
13. Educational curriculum
14. Hydration
15. Awareness activities
16. School canteen
17. Food safety and hygiene
18. Other-please specify: ___________________
    1. Who is responsible for the development of the nutrition program? (select all those that apply)
19. Nutritionists (individually or as part of an assigned committee)
20. Medical staff
21. Administrative staff
22. Academic staff
23. Parents
24. Other- please specify: ___________________
    1. What are the desired outcomes of the school nutrition program? (select all those that apply)
25. Improve students’ nutrition
26. Reduce and/ or prevent malnutrition
27. Reduce and/ or prevent obesity
28. Tackle health inequalities
29. Improve students’ academic performance
30. Improve students’ school attendance
31. Support parents and local community
32. Other- please specify: ___________________
    1. Is there a system in place to ensure the nutrition program is understood and implemented in practice by staff?
33. Yes
34. No
    1. What meals are covered by the nutrition program? (select all those that apply)
35. Breakfast
36. Lunch
37. Dinner
38. Snacks
39. None
    1. What grades does the nutrition program cover? (select all those that apply)
40. Kindergarten
41. Primary
42. Intermediate
43. Secondary
    1. Do you have a Monitoring and Evaluation plan for the implementation of the nutrition program?
44. Yes
45. No
    1. Are there defined indicators by which school nutrition program is monitored and evaluated?
46. Yes
47. No
    1. Are you aware of the school nutrition guidelines in Dubai?
48. Yes
49. No
    1. Is your school planning to implement any nutrition program in the next 3 – 5 years?
50. Yes
51. No

**Thank you for taking the time to respond to this survey.**
